# The relationship between a series of inflammatory markers on the risk of heart failure in different gender groups: Analysis from NHANES 2015–2018

**DOI:** 10.1371/journal.pone.0296936

**Published:** 2024-03-25

**Authors:** Ting Cheng, Dongdong Yu, Xingying Qiu, Wenwei OuYang, Geng Li, Li Zhou, Zehuai Wen

**Affiliations:** 1 Second Clinical College of Guangzhou University of Chinese Medicine, Guangzhou, China; 2 First Affiliated Hospital of Anhui University of Chinese Medicine, Hefei, China; 3 Guangdong Provincial Hospital of Chinese Medicine (Second Affiliated Hospital of Guangzhou University of Chinese Medicine), Guangdong Provincial Academy of Chinese Medical Sciences, Guangzhou, China; 4 Science and Technology Innovation Center of Guangzhou University of Chinese Medicine, Guangzhou, China; Xi’an Jiaotong University Medical College First Affiliated Hospital Department of Medical Oncology, CHINA

## Abstract

**Background:**

A better understanding of the level-grade inflammation for the development and worsening of heart failure (HF) in different gender groups is an unmet need. We performed an updated analysis on the impact of a series of systemic inflammation markers on HF.

**Methods:**

This compensatory cross-sectional study enrolled participants from the National Health and Nutrition Examination Survey (NHANES) 2015–2018. HF was based on the self-reported questions. Univariate and multivariate logistic regression were used to investigate the association between systemic immune-inflammation index (SII), high sensitivity C-reactive protein (hs-CRP), lymphocyte-to-monocyte ratio (LMR), neutrophil-to-lymphocyte ratio (NLR), platelet-to-lymphocyte ratio (PLR) and HF. For patients of different genders, *P* for trend was used to analyze potential linear trend relationships and the restricted cubic splines (RCS) were used to describe non-linear relationships. The additive interaction was evaluated by the relative excess risk due to interaction (RERI), attributable proportion (AP), and the synergy index (SI). The multiplicative interaction was evaluated by odds ratio (OR) and 95% confidence interval (CI) of product-term.

**Results:**

A total of 5,830 participants from the NHANES database were divided into two groups: the HF group (n = 210) and the non-HF group (n = 5620). After gender stratification, hs-CRP (OR: 1.01, 95% CI: 1.00–1.03), SII (OR: 1.00, 95% CI: 1.00–1.01), NLR (OR: 1.22, 95% CI: 1.11–1.35) and LMR (OR: 0.79, 95% CI: 0.65–0.93) were independent meaningful factors for HF in males, there was no non-linear relationship between the three factors (SII, NLR, hs-CRP, all *P* for non-linear > 0.05) and the prevalence of HF, but we detected a non-linear relationship between LMR and the prevalence of HF in males (*P* for non-linear < 0.05). An additive interaction of hs-CRP and NLR on the risk of HF in males (RERI (OR): 0.67, 95% CI: 0.12–1.34; AP (OR): 0.14, 95% CI: 0.02–0.24; SI (OR): 1.22, 95% CI: 1.03–1.44).

**Conclusions:**

In summary, hs-CRP, NLR, and LMR are superior meaningful markers for HF in males. SII may be a meaningful systemic inflammation warning marker for HF, which needs to be discriminated against with caution. Only detected a non-linear relationship between LMR and the prevalence of HF in males. NLR and hs-CRP may have an additive interaction in the prevalence of male HF patients. The outcome compensated for previous studies that still needed more studies for validation.

## Introduction

Heart failure (HF) is an emerging epidemic affecting more than 64.3 million patients worldwide due to its high hospitalization and mortality rates [1]. The inflammatory response potentially plays a role in the pathogenesis and adverse outcomes of HF due to abnormalities in the inflammatory cascade [2]. Previous studies have demonstrated evidence from humans that C-reactive Protein (CRP), neutrophil-to-lymphocyte ratio (NLR), platelet-to-lymphocyte ratio (PLR), and lymphocyte-to-monocyte ratio (LMR) are routinely available and emerging biomarkers that play important roles in HF-related inflammation, remodeling and fibrosis [3–5].

Moreover, recent studies have highlighted an emerging practical systemic immune-inflammation index (SII) that is related to cardio-cerebrovascular diseases (such as atrial fibrillation, cardiomyopathy, cerebrovascular disease and peripheral arterial vascular disease) [6, 7] as well as other disorders (such as cancer and diabetic depression) [8, 9]. However, the association between SII and HF has not been confirmed until now, and previous studies have mainly focused on the relationship between CRP and HF, rather than high-sensitivity C-reactive protein (hs-CRP). Furthermore, no research has investigated the prognostic role and dose-response relationship of SII, hs-CRP, NLR, PLR, and LMR in a large population of patients with HF simultaneously. A consensus still has not been reached regarding how a series of inflammation levels impact the risk of HF. Therefore, we report a large-scale epidemiological analysis among participants in the US National Health and Nutrition Examination Survey (NHANES) to better understand the associations between the five inflammatory indicators and HF prevalence in different gender groups. This study is based on widely available methods with a non-intrusive methodology, simple accessibility, and low cost. A better understanding of the level-grade inflammation for the development and worsening of HF is an unmet need that can provide new insights for earlier prevention and more innovative management.

## Methods

### Study population

**Fig 1:**
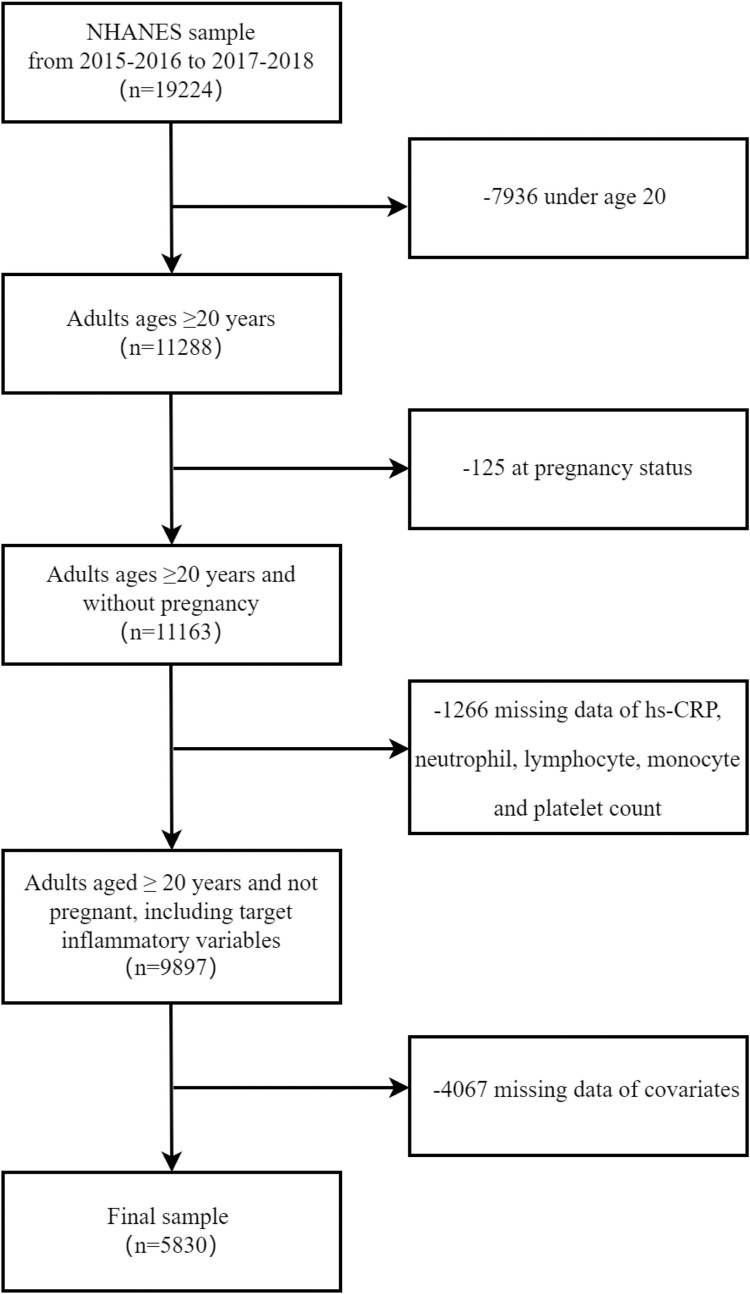
Flow chart of the screening process for the selection of eligible participants.

The present study utilized data from a cross-sectional study of the NHANES, which is designed to evaluate the health and nutritional status of the United States (US) population [10]. The data was from two 2-year NHANES survey cycles: 2015 to 2016 and 2017 to 2018. The research ethics review board at the National Center for Health Statistics (NCHS) approved the survey protocol. All of the participants completed the NHANES survey and signed an informed consent. The present study was based on a retrospective analysis, which lacked personal identifiers, and ethics committee approval and informed consent were not required. Additional comprehensive information regarding the NHANES can be found at https://wwwn.cdc.gov/nchs/nhanes/Default.aspx. The exclusion criteria included: (a) age < 20 years; (b) pregnancy status; (c) missing data of HF; (d) missing data of neutrophil, lymphocyte, monocyte and platelet count; (e) missing data of covariates. Ultimately, 5830 participants were included in the final analysis (Fig 1).

### Assessment of heart failure

The verification of HF was based on the questionnaire (MCQ.J.xpt) by asking “Ever told had congestive heart failure?”, the participants who reported “Yes” to the question indicated a history of diagnosed HF.

### Systemic inflammation markers

As reported by the laboratory method files section in NHANES, hs-CRP (mg/L) reagent was based on the highly sensitive near-infrared particle immunoassay rate methodology and measured on the Beckman Coulter UniCel DxC 600 and 660i synchron access chemistry analyzers. The complete blood cell count (CBC) via a automated hematology analyzing devices (Coulter® DxH 800 analyzer) to derives CBC parameters [11]. Calculation were as follows [12]: SII=(neutrophils×platelets)/lymphocytes,NLR=neutrophils/lymphocytes,PLR=platelets/lymphocytes, and LMR=lymphocytes/monocytes.

### Covariates definition

The following factors were included in the statistical model as covariates. Socio-demographic characteristics were defined, including age, gender, race, body mass index (BMI), marital status, education levels, job, health insurance, family poverty income ratio (PIR), and survey released cycle (Year). PIR was calculated by dividing family income by the poverty guidelines, where a smaller ratio indicates a lower income. In addition, a family history of heart attack, diabetes, asthma, arthritis, gout, stroke, hypertension, and trouble sleeping was based on questionnaire sections by asking “Ever told you had XX?” and recorded by “Yes/No”. Smoking is dichotomized to “Yes/No” by asking “Smoked at least 100 cigarettes in life?”. Drinking is dichotomized to “Yes/No” by asking “Ever have 4/5 or more drinks every day?”. This investigation included the aforementioned covariates that may affect the relationship between systemic inflammation markers and the prevalence of HF.

### Statistical analyses

The baseline characteristics of the included participants were divided into non-HF and HF groups, and continuous variables were analyzed and reported as mean ± standard deviation (SD) or median with inter quartile range (IQR), while categorical variables were analyzed by counting number (percentages) and treated by one-way analysis of variance (ANOVA). Considering that blood inflammatory markers often influence each other, we first conducted a correlation analysis between the inflammatory markers. The significant risk-related variables of univariate and multivariate analysis for HF were based on the R packages ‘readr’ (readr 2.1.4), ‘plyr’ (version 1.8.8), ‘car’ (version 3.1–2) to calculate odds ratios (ORs) and corresponding 95% confidence intervals (CIs). The individual variables of hs-CRP, NLR, LMR, and SII were included separately for each model, based on two groups with different adjustments, and adjusted for all the significant covariates shown in the final model. The diagnosis of multicollinearity between covariates was fully considered using the variance inflation factor (VIF) in a multiple regression model. A trend test (*P* for trend) was used to analyze the trend relationship between the target variable and the outcome. Further stratified logistic regression analysis was conducted to identify variables that modify the association in gender-stratified subgroups. A restricted cubic spline (RCS) was used to explore the non-linear relationship with 5 knots selected to smooth the curve [13]. The additive interaction between hs-CRP and NLR on the risk of HF was evaluated by the relative excess risk due to interaction (RERI), attributable proportion (AP) and the synergy index (SI) [14]. When the 95% CI of RERI or AP was not included 0, or the 95% CI of SI did not contain 1, we considered there was an additive interaction. The multiplicative interaction between hs-CRP and NLR on the risk of HF was evaluated by OR, when the 95% CI of product-term was not contained 1, we considered there was a multiplicative interaction. Although the aim of this study was to explore correlation rather than causality, OR can still be used to describe the extent of correlation described above.

A value of *P* < 0.05 (two-sided) was considered statistically significant. SPSS 18.0 (IBM Corporation, Armonk, NY, USA) and R version 4.3 (the R Foundation for Statistical Computing, Vienna, Austria) were used for statistical analysis. The included variables were considered in our analyses due to their reported diagnostic value for cardiovascular diseases. Although NHANES is a complex sampling design, any missing values in the included variables have been excluded. Weight analysis was also not used in this study.

## Results

### Baseline characteristics of participants

A total of 5,830 participants were involved in Table 1 (mean age, 50.2 years old; 52.5% male) and 3.6% participants were categorized as having HF. There were statistically significant differences in age, gender, race, education, marital status, job, BMI, health insurance, PIR, drinking, smoking, family history of heart attack, diabetes, arthritis, gout, hypertension, trouble sleeping, asthma, and stroke between patients with HF and those without (all *P* < 0.01). HF is more common in the elderly, male, obesity, low educated, no spouse, uninsured, and impoverished individuals. The survey released cycle (Year) did not differ between patients with and without HF (*P* > 0.05). Compared to those without HF, hs-CRP, SII, and NLR were at higher levels in people with HF (all *P* < 0.01). LMR was at lower levels in people with HF (*P* < 0.01). No significant difference was found in the PLR levels between the non-HF and HF groups (*P* > 0.05). OR and its 95% CI of covariates can be found in Table 2. The correlation heatmap of inflammation markers is shown in S1 Fig. SII and NLR are strongly correlated (r = 0.87), while the correlation strength between other markers is not high.

**Table 1:** Baseline characteristics of participants, NHANES 2015–2018 (n = 5,830).

	Non-HF	HF	*P*
	(n = 5620)	(n = 210)	
Age^a^	49.6 ± 17.3	67.5 ± 10.6	<0.001
Year^b^			0.880
2015–2016	3321 (96.4)	123 (3.6)	
2017–2018	2299 (96.4)	87 (3.6)	
Gender^b^			0.001
Male	2928 (95.6)	134 (4.4)	
Female	2692 (97.3)	76 (2.7)	
Race^b^			0.005
Mexican American	878 (98.2)	16 (1.8)	
Other Hispanic	588 (95.6)	27 (4.4)	
Non-Hispanic	4154 (96.1)	167 (2.9)	
BMI (kg/m^2^) ^b^			<0.001
<25	878 (98.2)	16 (1.8)	
25–29.9	588 (95.6)	27 (4.4)	
≧30	4154 (96.1)	167 (3.9)	
Education^b^			<0.001
Less than high school graduate or equivalent	2338 (95.2)	119 (4.8)	
College graduate or above	3282 (97.3)	91 (2.7)	
Marital status^b^			0.017
Married/ living with partner	3378 (96.9)	109 (3.1)	
Widowed/divorced/separated/never married	2242 (95.7)	101 (4.3)	
Health insurance^b^			0.001
Yes	4702 (96.0)	194 (4.0)	
No	918 (98.3)	16 (1.7)	
Job^b^			<0.001
Government employee	529 (99.2)	4 (0.8)	
Business/professional practice/ farm	2874 (99.0)	30 (1.0)	
Unknow	2217 (92.6)	176 (7.4)	
PIR^a^	2.59 ± 1.61	1.89 ± 1.32	<0.001
Close relative history of heart attack^b^			0.009
Yes	2992 (95.8)	131 (4.2)	
No	2628 (97.1)	79 (2.9)	
Drinking^b^			<0.001
Yes	907 (92.5)	74 (7.5)	
No	4713 (97.2)	136 (2.8)	
Smoking^b^			<0.001
Yes	2688 (94.8)	148 (5.2)	
No	2932 (97.9)	62 (2.1)	
Diabetes^b^			<0.001
Yes	800 (89.9)	90 (10.1)	
No	4820 (97.6)	120 (2.4)	
Asthma^b^			<0.001
Yes	839 (92.6)	67 (7.4)	
No	4781 (97.1)	143 (2.9)	
Arthritis^b^			<0.001
Yes	1538 (92.5)	124 (7.5)	
No	4082 (97.9)	86 (2.1)	
Gout^b^			<0.001
Yes	290 (87.6)	41 (12.4)	
No	5330 (96.9)	169 (3.1)	
Stroke^b^			<0.001
Yes	194 (80.5)	47 (19.5)	
No	5426 (97.1)	123 (2.9)	
Hypertension^b^			<0.001
Yes	1965 (93.7)	132 (6.3)	
No	3655 (97.9)	78 (2.1)	
Trouble sleeping^b^			<0.001
Yes	1631 (92.9)	124 (7.1)	
No	3989 (97.9)	86 (2.1)	
Systemic inflammation markers^c^			
hs-CRP	1.9 (0.8, 4.4)	2.9 (1.3, 6.8)	<0.001
SII	440.1 (310.5, 620.2)	484.3 (326.9, 690.1)	0.041
NLR	1.9 (1.4, 2.5)	2.4 (1.7, 3.3)	<0.001
LMR	3.8 (3.0, 5.0)	2.9 (2.2, 3.6)	<0.001
PLR	111.2 (87.9, 139.5)	106.3 (88.1, 138.6)	0.700

NHANES: National Health and Nutrition Examination Survey; HF: heart failure; Year: survey released cycle; BMI: body mass index; SII: systemic immune-inflammation index; hs-CRP: high sensitivity C-reactive protein; NLR: neutrophil-to-lymphocyte ratio; PLR: platelet-to-lymphocyte ratio; LMR: lymphocyte-to-monocyte ratio; a: reported as mean ± standard deviation (SD); b: reported as counting number (percentages); c: reported as median (Q1, Q3).

**Table 2:** Results of univariable logistic regression analysis between covariates and HF.

	Univariable analysis		
Characteristics	OR	95% CI	*P*
Year	1.02	0.77–1.35	0.880
Gender	0.62	0.46–0.82	0.001
Age	1.08	1.06–1.09	<0.001
Race			
Other Hispanic	2.52	1.35–4.72	0.004
Non-Hispanic	2.21	1.31–3.70	0.003
Education	0.54	0.41–0.72	<0.001
Marital status	1.40	1.06–1.84	0.018
Job			
Business/professional practice/ farm	1.38	0.48–3.93	0.546
Unknow	10.50	3.88–28.42	<0.001
BMI (kg/m^2^)			
25–29.9	1.30	0.83–2.04	0.248
≥30	2.54	1.71–3.77	<0.001
Close relative history of heart attack	1.46	1.10–1.94	0.009
Health insurance	2.37	1.41–3.96	0.001
PIR	0.73	0.67–0.81	<0.001
Drinking	2.83	2.11–3.79	<0.001
Smoking	2.60	1.93–3.52	<0.001
Diabetes	4.52	3.40–6.00	<0.001
Asthma	2.67	1.98–3.60	<0.001
Arthritis	3.83	2.89–5.06	<0.001
Gout	4.46	3.11–6.40	<0.001
Stroke	8.06	5.66–11.5	<0.001
Hypertension	3.15	2.36–4.19	<0.001
Trouble sleeping	3.53	2.66–4.67	<0.001

HF: heart failure; Year: survey released cycle; BMI: body mass index; PIR: family poverty income ratio; OR: odds ratio; CI: confidence intervals.

### Systemic inflammation markers and HF risk

In the crude model (model 1 in Table 3), hs-CRP, NLR, LMR, and SII levels were both independent influencing factors for HF (*P*<0.001). A high level of hs-CRP was correlated to a higher prevalence of HF (OR: 1.03, 95% CI: 1.02–1.04, *P*<0.001). A high level of NLR was also correlated to a higher prevalence of HF (OR: 1.33, 95% CI: 1.23–1.43, *P*<0.001). A low level of LMR was correlated to a higher prevalence of HF (OR: 0.60, 95% CI: 0.53–0.70, *P*<0.001). A high level of SII showed little statistical significance in the prevalence of HF based on ORs, which was slightly larger than 1 (*P*<0.001). There was a significant linear relationship trend between LMR, hs-CRP, SII and NLR in the prevalence of HF (all *P* for trend < 0.05).

**Table 3:** Univariate and multivariate analyses between hs-CRP/SII/NLR/LMR and HF in different models.

	Model 1			Model 2		
	OR (95% CI)	*P*	*P* for trend	OR (95% CI)	*P*	*P* for trend
hs-CRP	1.03 (1.02–1.04)	< 0.001	< 0.001	1.03 (1.02–1.04)	< 0.001	< 0.001
SII	1.00 (1.00–1.01)	< 0.001	0.027	1.00 (1.00–1.01)	0.005	0.160
NLR	1.33 (1.23–1.43)	< 0.001	< 0.001	1.20 (1.10–1.30)	< 0.001	0.006
LMR	0.60 (0.53–0.67)	< 0.001	< 0.001	0.79 (0.70–0.89)	< 0.001	< 0.001
	Model 3			Model 4		
	OR (95% CI)	*P*	*P* for trend	OR (95% CI)	*P*	*P* for trend
hs-CRP	1.02 (1.01–1.03)	0.002	0.029	1.01 (1.00–1.02)	0.005	0.244
SII	1.00 (1.00–1.01)	0.011	0.307	1.00 (0.99–1.00)	0.185	0.841
NLR	1.20 (1.10–1.30)	< 0.001	0.014	1.15 (1.05–1.25)	0.002	0.134
LMR	0.79 (0.69–0.89)	< 0.001	< 0.001	0.82 (0.72–0.92)	0.001	< 0.001

HF: heart failure; NLR: neutrophil-to-lymphocyte ratio; hs-CRP: high sensitivity C-reactive protein; SII: systemic immune-inflammation index; LMR: lymphocyte-to-monocyte ratio; OR: odds ratio; CI: confidence intervals.

Model 1: hs-CRP, NLR, LMR, and SII were included separately for each analysis, adjusted for none.

Model 2: hs-CRP, NLR, LMR, and SII were included separately for each analysis, adjusted for age, gender, race.

Model 3: hs-CRP, NLR, LMR, and SII were included separately for each analysis, adjusted for age, gender, race, education, marital status, job, BMI, health insurance, PIR, drinking, smoking, close relative history of heart attack.

Model 4: hs-CRP, NLR, LMR, and SII were included separately for each analysis, adjusted for age, gender, race education, marital status, job, BMI, health insurance, PIR, drinking, smoking, close relative history of heart attack, diabetes, arthritis, gout, hypertension, trouble sleeping, asthma, and stroke.

We evaluated the correlation between each variable of hs-CRP, NLR, LMR, and SII and HF using multivariate logistic regression analysis with adjusting some covariates for each analysis (model 2–4 in Table 3). In all models, hs-CRP, NLR, and LMR were meaningful independent influencing factors for the prevalence of HF (all *P*<0.01). SII has no statistical significance in Model 4 (*P* = 0.185), and we found that the history of other diseases can affect the relationship between SII and the prevalence of HF. Comparing the four models, LMR exhibited a significant linear relationship trend with the prevalence of HF (all *P* for trend<0.001), but inconsistency in trend test results of hs-CRP, NLR, and SII among different models.

### Gender-stratified analyses

Two models were used to evaluate the correlation between hs-CRP, SII, NLR, LMR and HF in gender-stratified models (Table 4). In the crude model, hs-CRP, NLR, and LMR were meaningful independent influencing factors for the prevalence of HF (all *P*<0.05), and the higher level of NLR and hs-CRP were correlated with a higher prevalence of HF in males (*P* for trend < 0.001). However, the level of LMR was negatively correlated with the prevalence of HF (*P* for trend < 0.001) in both males and females.

**Table 4:** Gender-stratified logistic regression model by multivariate analyses.

	Model 1			Model 2		
	OR (95% CI)	*P*	*P* for trend	OR (95% CI)	*P*	*P* for trend
Male						
hs-CRP	1.04 (1.02–1.05)	< 0.001	< 0.001	1.01 (1.00–1.03)	0.018	0.067
SII	1.00 (1.00–1.01)	< 0.001	< 0.001	1.00 (1.00–1.01)	0.018	0.558
NLR	1.37 (1.25–1.50)	< 0.001	< 0.001	1.22 (1.11–1.35)	< 0.001	0.030
LMR	0.57 (0.48–0.67)	< 0.001	< 0.001	0.79 (0.65–0.93)	0.006	0.002
Female						
hs-CRP	1.01 (1.00–1.03)	0.011	0.085	1.02 (0.99–1.03)	0.854	0.721
SII	1.00 (0.99–1.01)	0.451	0.620	1.00 (0.99–1.01)	0.680	0.337
NLR	1.20 (1.06–1.38)	0.004	0.149	1.01 (0.86–1.17)	0.854	0.750
LMR	0.66 (0.55–0.79)	< 0.001	< 0.001	0.87 (0.72–1.03)	0.125	0.026

HF: heart failure; NLR: neutrophil-to-lymphocyte ratio; hs-CRP: high sensitivity C-reactive protein; SII: systemic immune-inflammation index; LMR: lymphocyte-to-monocyte ratio; OR: odds ratio; CI: confidence intervals.

Model 1: hs-CRP, NLR, LMR, and SII were included separately for each analysis, adjusted for none.

Model 2: hs-CRP, NLR, LMR, and SII were included separately for each analysis, adjusted for age, race, education, marital status, job, BMI, health insurance, PIR, drinking, smoking, close relative history of heart attack, diabetes, arthritis, gout, hypertension, trouble sleeping, asthma, and stroke.

In model 2, we did not find that hs-CRP, SII, NLR and LMR were independent influencing factors for HF in females (all *P*>0.05 and *P* for trend>0.05). On the contrary, the relationship between hs-CRP, SII, NLR and LMR and the prevalence of HF remains significant in males (all *P* < 0.05), but only NLR and LMR exhibits a significant linear relationship trend with the prevalence of HF (all *P* for trend < 0.05).

### RCS analysis of hs-CRP/SII/NLR/LMR and HF risk

**Fig 2:**
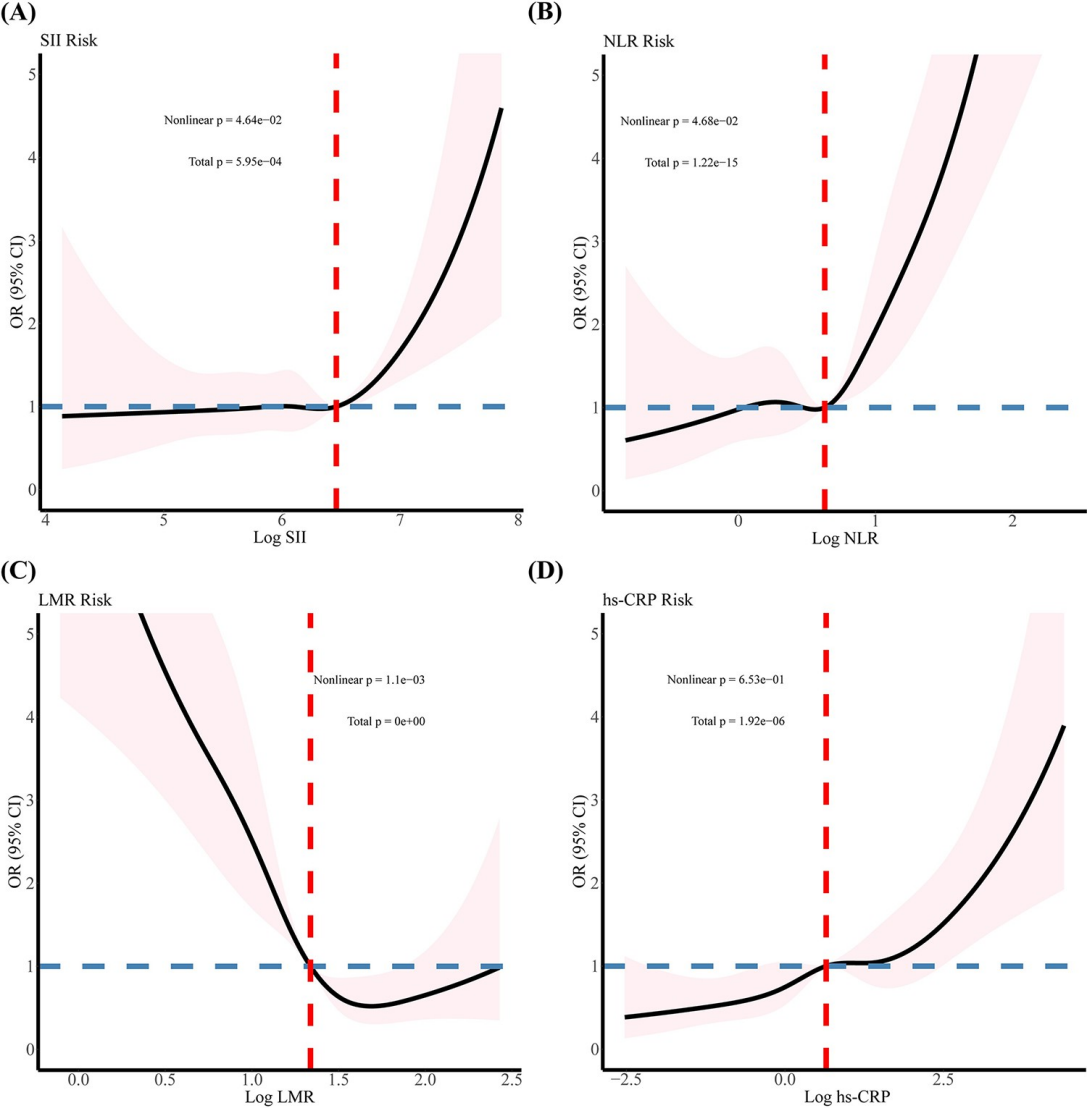
The analysis of restricted cubic spline regression. (A-D adjusted for none, hs-CRP, SII, NLR, LMR enter into the model as log (original value)).

**Fig 3:**
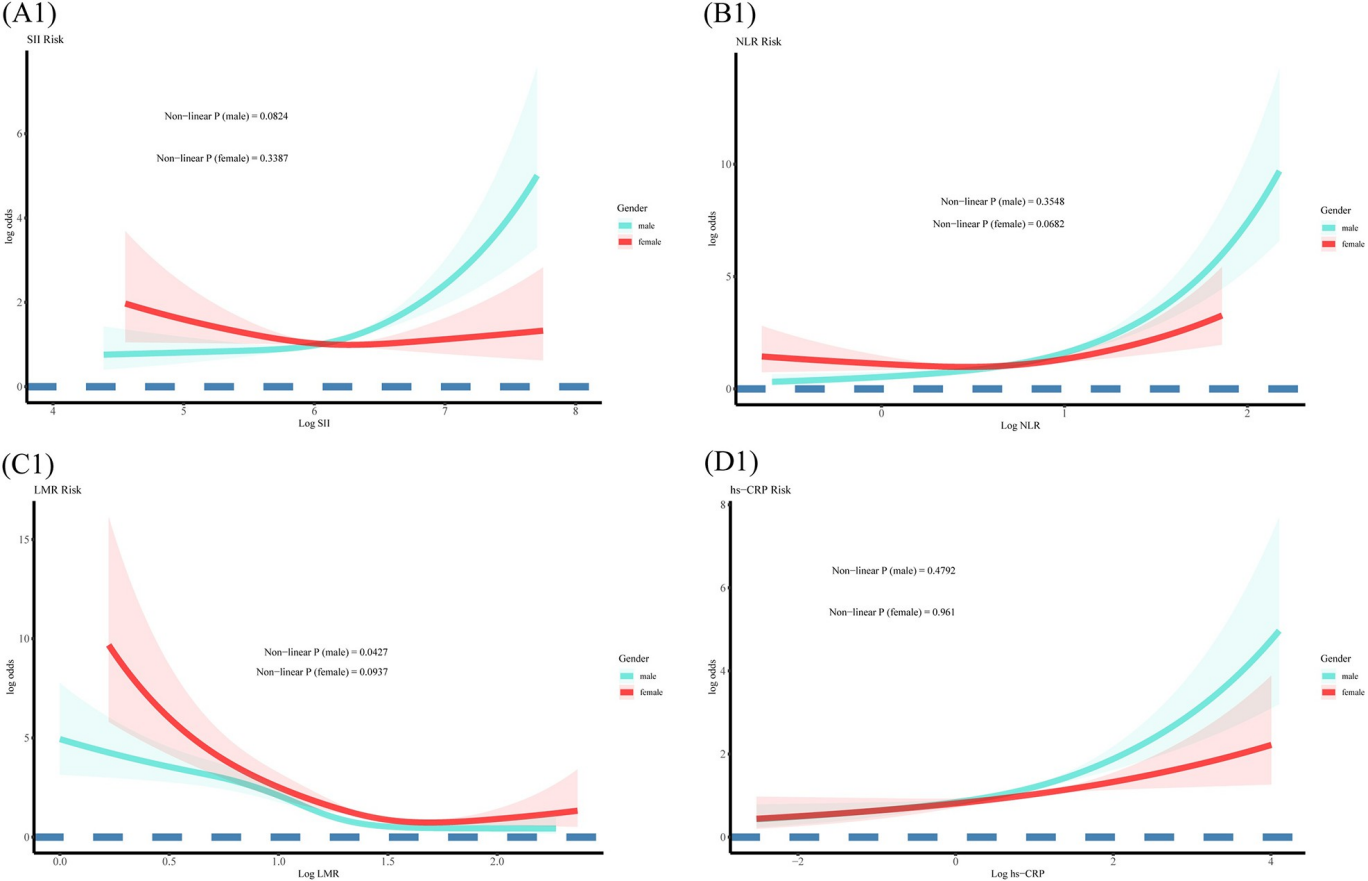
The analysis of restricted cubic spline regression. (A1-D1 after gender stratified, hs-CRP, SII, NLR, LMR enter into the model as log (original value)).

In restricted cubic spline regression (Figs 2 and 3), Fig A showed the trend of an OR for HF with increasing SII (*P* for non-linear < 0.05), which did not remained consistent across sex subgroups (all *P* for non-linear > 0.05, Fig 3A1). Fig B shows the trend of a fast-increasing OR for HF with increasing NLR (*P* for non-linear < 0.05), which exhibited a stronger trend in the male group (all *P* for non-linear > 0.05, Fig 3B1). Fig D demonstrates the trend of an increasing OR for HF with increasing hs-CRP (*P* for non-linear > 0.05), which also remained consistent across sex subgroups (all *P* for non-linear > 0.05). In summary, we detected a significant non-linear relationship between LMR and the prevalence of HF in Fig C (*P* for non-linear < 0.01), but only in males after gender stratification (*P* for non-linear < 0.05, Fig 3C1).

### RERI analyses

As shown in Table 5, we established the interaction term of hs-CRP and NLR to assess the additive interaction in different genders. We observed an additive interaction of hs-CRP and NLR on the risk of HF (RERI: 0.67, 95% CI: 0.12–1.34; AP: 0.14, 95% CI: 0.02–0.24; SI: 1.22, 95% CI: 1.03–1.44), which indicated that there might be a synergistic effect, but it does not exist in the female population. Simultaneously, we also assessed a multiplicative interaction of hs-CRP and NLR on the risk of HF, and we found that the multiplicative interaction was not statistically significant in both genders. So, we indicated that there might be a synergistic effect only in males.

**Table 5:** The additive and multiplicative interaction of hs-CRP and NLR on the risk of HF.

	Variables	OR (95% CI)	*P*
Male			
	hs-CRP	1.39 (1.02–1.90)	
	NLR	3.61 (2.31–5.62)	
	hs-CRP *NLR	0.93 (0.72–1.21)	0.590
	RERI	0.67 (0.12–1.34)	
	AP	0.14 (0.02–0.24)	
	SI	1.22 (1.03–1.44)	
Female			
	hs-CRP	1.39 (1.00–1.96)	
	NLR	1.69 (0.82–3.47)	
	hs-CRP *NLR	0.87 (0.58–1.30)	0.504
	RERI	-0.03 (-0.89–0.40)	
	AP	-0.02 (-0.38–0.22)	
	SI	0.97 (-0.58–2.47)	

HF: heart failure; NLR: neutrophil-to-lymphocyte ratio; hs-CRP: high sensitivity C-reactive protein; OR: odds ratio; CI: confidence intervals; RERI: relative excess risk due to interaction; AP: attributable proportion; SI: synergy index. Adjusted for none. NLR and hs-CRP enter into the model as log(original value).

## Discussion

This present cross-sectional study found some interesting findings. Firstly, there were positive associations between hs-CRP and NLR and the prevalence of HF overall. However, SII was possibly a marker of systemic inflammation in HF patients, LMR is negatively correlated with the prevalence of HF. Secondly, hs-CRP, SII, NLR and LMR were independent influencing factors for HF among males, but we need to be carefully determined in female HF patients. In addition, we only detected a non-linear relationship between LMR and the prevalence of HF in males. In addition to contributing to the most significant ORs and having the widest generality in sex subgroups, NLR might be the best marker for HF risk. Finally, an additive interaction of hs-CRP and NLR on the risk of HF in males was found.

It is gradually becoming more broadly recognized that systemic inflammation initiates and aggravates the pathological process of chronic diseases. A plethora of inflammatory predictors associated with the risk of cardiovascular diseases (CVDs) were discovered and targeted treatments were proposed. One study investigated the inflammatory scores of monocyte-to-lymphocyte ratio (MLR), NLR, and PLR, which are associated with the severity of disease in HF [15]. CRP is the most investigated marker of all-cause mortality in HF [16] and has been identified as a risk marker of post-infarct ventricular dysfunction [17]. A large sample analysis showed that CRP level > 5 mg/L effectively predicted postoperative HF, and NLR > 3.5 had a good predictive effect on all-cause mortality within 30 days after surgery [18]. Moreover, NLR is also associated with common heart conditions, high NLR levels at admission in patients with acute heart failure (AHF) are independently associated with major cardiovascular events (MACE). A study found that NLR can be used to predict the development of atrial fibrillation in diabetes patients [19]. A high NLR in conjunction with neutrophil transcriptional activation is associated with systemic inflammation and functional impairment in HF with preserved ejection fraction (HFpEF) [4].—Liu Z established a novel nomogram integrated with LMR (HR: 0.85, 95% CI: 0.78–0.93) and PLR (HR: 1.002, 95% CI: 1.000–1.004) to evaluate the risk of cardiovascular readmission or all-cause mortality in patients with CHF [20]. The above are consistent with our research results and verified synchronously in the same large sample population based on NHANES data. However, a Mendelian randomization (MR) study did not identify convincing evidence that CRP was causally linked to HF [21]. The reason for inconsistency with our conclusion may be due to samples and test methods. Therefore, we need to conduct additional human and animal studies to confirm our findings.

In recent years, the relationship between SII and inflammatory diseases and cancer has received attention due to its integrated immunity and inflammation indicators to reflect the state of the body. The latest study confirms that SII level is in direct proportion to the degree of diabetes kidney damage in patients with type 2 diabetes [22]. A study suggests that SII is a risk factor for patients with acne vulgaris, but cannot predict disease severity grading [23]. Similarly, studies have found that SII is a risk factor for liver steatosis, but it is not associated with liver fibrosis [24]. Meta-analysis showed that high SII levels significantly reduced overall survival (OS) in patients with small-cell lung cancer and glioblastoma [25, 26]. In addition, in recent years, some studies have begun to focus on the relationship between SII and cardiovascular disease. The study investigated the association between SII and system inflammation response index (SIRI) with all-cause mortality and cardiovascular mortality in America over a 20-year follow-up period [27]. Zhu Y—confirmed that SII was an independent indicator of clinical prognosis in acute ST-elevation myocardial infarction (STEMI) patients [28]. The Dongfeng-Tongji cohort study in Chinese suggested SII may serve as a useful marker to elucidate the role of the interaction of thrombocytosis, inflammation and immunity in the development of stroke and coronary heart disease (CHD) in the middle-aged and elderly [29]. SII is positively correlated with the severity of coronary angiography in patients with CHD [30] and has a better predictive ability for MACE [31]. Obtaining information due to the heterogeneity of individual populations, SII may be an independent risk factor for HF, and the impact was significantly weakened among female populations in our study.

However, the precise immunologic mechanisms underlying a series of inflammatory markers that increase the risk of developing HF are not completely understood, but some research results have been recognized. Leukocyte recruitment from the blood to tissues is a hallmark of inflammation and antimicrobial host defense [32]. Neutrophils, monocytes, lymphocytes and platelets play different roles in the inflammatory cascade reaction. Vascular inflammation underlies most forms of cardiovascular disease (HF, stroke, etc.) [33]. Neutrophils are immune cells with potent antimicrobial properties [34] and the interaction between neutrophils and endothelial cells for the pathogenesis of vascular inflammation is critical. Inflammation causes a decrease in the absolute lymphocyte count in the human body due to an increase in NLR [35], and the abnormal rise of platelets may be caused by it [36]. Under inflammatory conditions that do not induce major disruption to vascular structure, individual platelets support the recruitment of leukocytes to sites of inflammation, mobilized to the vessel wall where they interact with leukocytes and appear to seal gaps that arise between endothelial cells [37]. Platelets are able to interact with a large variety of cell types (leukocytes, endothelial cells, etc.) carry a highly inflammatory payload and are able to transport, synthesize, and deposit cytokines, chemokines, and lipid mediators. Thus, atherosclerotic cardiovascular disease, stroke, peripheral arterial disease, and other malignant clinical events can be induced and propagated [38]. We speculate that the relationship between inflammatory indicators and sex steroid hormones in the male population deserves further research.

The NHANES data were selected using a complex, multi-stage probability sampling method for a representative sample of the civilian noninstitutionalized resident population to ensure high data quality. Our study obtained relatively reliable results based on the elimination of missing data. More importantly, with consistent adjustments, we simultaneously examined the specific impacts of four systemic inflammation markers on HF prevalence in the same population and probably avoided unstable results due to heterogeneity between different studies. Unlike other studies, we not only analyzed the influence on the entire population, but we conducted multiple statistical model analyses mainly stratified by sex. This study was explored the association between blood inflammatory markers and HF in different genders subgroups and in the same large population. Therefore, the findings have additional public health implications in the prevention of HF.

In our study, there are some limitations that are inevitable. As a result of employing a cross-sectional design, it can only provide information on the association between the relevant biomarkers and HF, but cannot establish causality. Secondly, because only baseline CBC counts are available, we were unable to assess the prevalence of HF based on fluctuations in inflammation ratios. In spite of this, our results were generally in line with those of previous studies. Thirdly, the samples in this study are all from the European population, and the results may not be applicable to non-Europeans and may affect the results due to incomplete consideration of weights. Fourthly, due to the inability to directly obtain clinical records or conduct detailed medical evaluations in existing cross-sectional studies, we can only rely on participant’s self-reports to define the occurrence of HF, which may lead to some missed and misdiagnosed cases. Despite our efforts to adjust for confounding factors, the limited data from NHANES did not account for the use of antibiotics, immunosuppressive medications, and the impact of cardiovascular ultrasound-related data. In addition, this study did not develop a risk score based on these inflammatory biomarkers to evaluate its relationship with HF. The next research direction is to conduct a comprehensive risk assessment based on different databases. Thus, the study’s shortcomings call for cautious interpretation of the findings in clinical practice. Therefore, it is still necessary to conduct further research by randomized controlled trials (RCTs) in different large-scale groups to determine the causal relationship.

## Conclusions

We suggest that hs-CRP, NLR, and LMR are superior meaningful markers for HF in males, and NLR might be the best one for the prevalence of HF regardless of gender. SII may be a meaningful systemic inflammation warning marker for HF, which needs to be discriminated against with caution. Only detected a non-linear relationship between LMR and the prevalence of HF in males. NLR and hs-CRP may have an additive interaction in the prevalence of male HF patients. In the future, the causal relationship and precise mechanisms of the association between hs-CRP, SII, NLR, LMR and HF should be investigated.

## Supporting information

S1 Fig The correlation heatmap of four inflammation markers. (TIF)
