# Supplementary figures and images for: The relationship between a series of inflammatory markers on the risk of heart failure in different gender groups: Analysis from NHANES 2015–2018
Source: PLoS One. 2024 Mar 25;19(3):e0296936. doi: 10.1371/journal.pone.0296936 (PMC10962816; doi:10.1371/journal.pone.0296936)

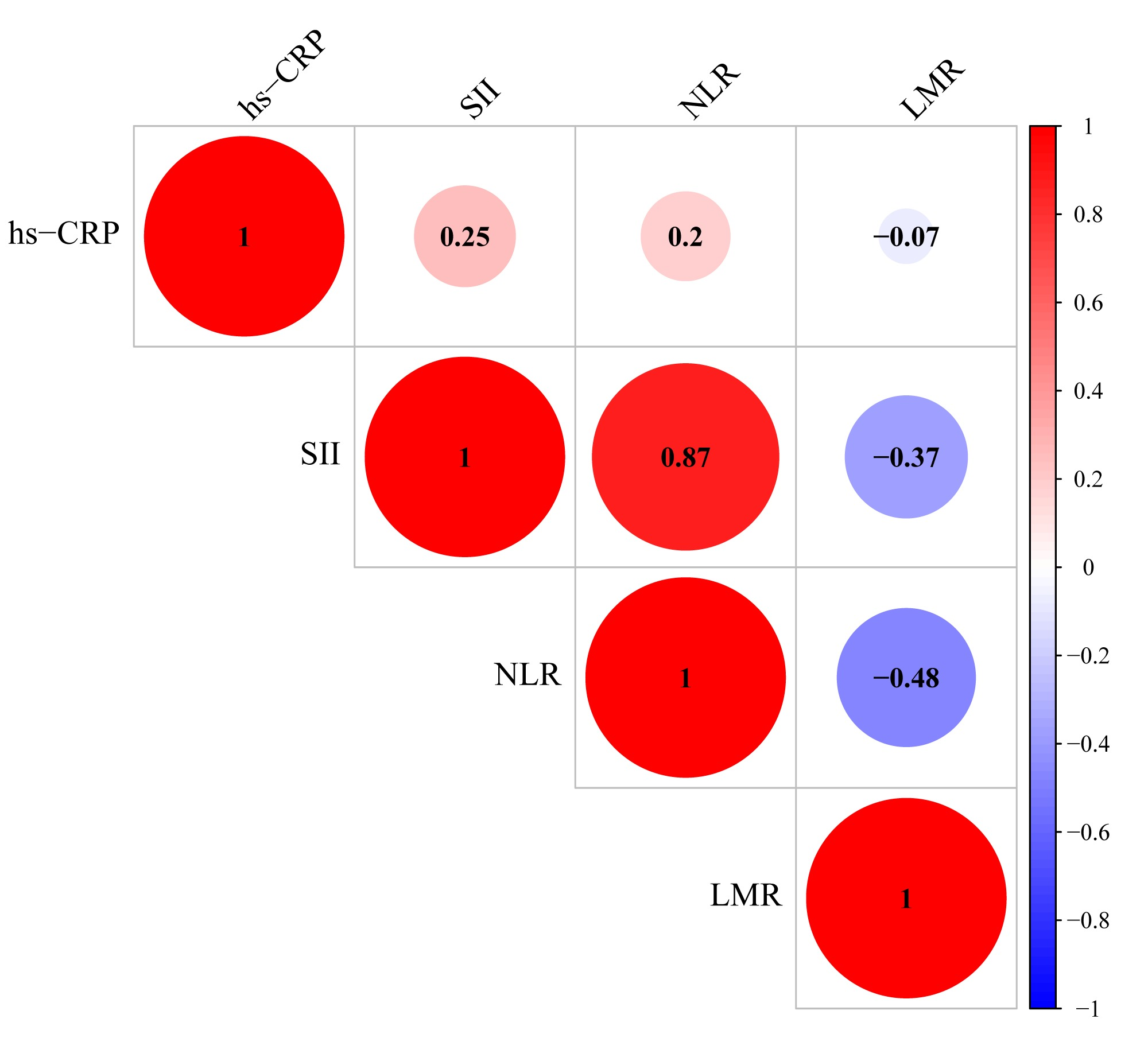

Supplement: S1 Fig — (TIF) [file pone.0296936.s001.tif]
